# The potential impact of intraoperative neurophysiological monitoring on neurological function outcomes after postnatal spina bifida repair

**DOI:** 10.1007/s00381-025-06778-5

**Published:** 2025-02-24

**Authors:** Matthias Krause, Florian Leibnitz, Matthias Manfred Knüpfer, Andreas Merkenschlager, Christoph J. Griessenauer, Janina Gburek-Augustat

**Affiliations:** 1 Department for Neurosurgery, University Hospital Leipzig, Leipzig, Germany; 2 Department for Neonatology, University Hospital Leipzig, Leipzig, Germany; 3 Division of Neuropediatrics, Department for Children’S and Adolescence Health, University Hospital Leipzig, Leipzig, Germany; 4 Dept. of Neurosurgery and Pediatric Surgery, Pediatric Neurosurgery, Christian Doppler Klinik, University Hospital Salzburg, Paracelsus Medical University, Müllner Hauptstrasse 48, Salzburg, Austria

**Keywords:** Myelomeningocele, Intraoperative monitoring, Neurophysiology, Two-hit theory

## Abstract

**Introduction:**

The management of open neural tube defects (ONTD) has significantly improved with fetal surgery, but many children remain ineligible for fetal therapy. This study assesses the impact of intraoperative neurophysiological monitoring (IONM) during postnatal myelomeningocele (MMC) repair and its potential to optimize functional outcomes.

**Patients and methods:**

Seven newborns with thoracolumbar or lumbar MMC underwent postnatal surgical repair using IONM. Neuromonitoring included motor-evoked potentials (MEP), sensory-evoked potentials (SEP), electromyography (EMG), and bulbocavernosus reflex (BCR). Preoperative neurological assessments were compared with postnatal outcomes at 2 years, along with anatomical levels on MRI and IONM results.

**Results:**

At birth, 6 of 7 newborns showed better functional levels than expected by the anatomical level of the ONTD in fetal MRI. Intraoperative EMG responses were normal in all but one patient, but only 30% of patients had normal MEP and SEP responses. IONM was useful to detect undue mechanical stress during ONTD repair surgery and intraoperative identification of functional nerve roots. Neurological function deteriorated in the early postoperative period but stabilized at a level above the anatomical MRI level in 85% of the patients by 2 years of age.

**Conclusion:**

IONM in postnatal ONTD repair is a safe and valuable tool that has the potential to increase surgical safety by detecting undue mechanical stress to the neural structure. Standardized postnatal management strategies, including the use of IONM, should be refined. Further studies are necessary to evaluate the prognostic value of EMG during postnatal surgery and its potential to result in better long-term outcome by preventing additional intraoperative damage.

## Introduction

Myelomeningocele and other open neural tube defects (ONTD) pose a significant risk for sensory and motor deficits. Over the past two decades, advances in fetal surgery have shifted the treatment paradigm for these defects [1–4]. However, not all patients are eligible for fetal surgery, making postnatal repair an essential treatment option.

Intraoperative neurophysiological monitoring (IONM) has been used extensively in spinal surgery, including for spinal malformations. IONM assists in anatomical orientation and identification of functional neural structures within the placode during surgery. Eibach and Pang recently advocated for the routine use of IONM during postnatal spina bifida surgery, highlighting its role in preserving function by guiding more precise resections [5].

This study aims to evaluate the use of IONM in postnatal MMC repair and to correlate intraoperative electrophysiological findings with neurological outcomes and radiological levels of ONTD. Furthermore, the study explores the distinction between preoperative deficits and those potentially caused by the postnatal repair process.

## Patients and methods

Between 2016 and 2021, seven newborns with open lumbar or thoracolumbar myelomeningocele underwent postnatal surgical repair using IONM. This cohort was selected from 18 newborns based on IONM availability and preoperative assessment. An overview of all patient demographics is shown in Table 1.

**Table 1:** Overview of the patients and their spina bifida anatomical characteristics, intraoperative neurophysiological monitoring during primary closure, and follow-up outcome. The motor level indicates the highest level of full-strength motor innervation

Patient no	Sex	Anatomical level in MRI	Postpartal preOP motor level	IONM during primary NTD closure				Follow-up motor outcome level	Difference of motor function level with anatomical level
				MEP	SEP	BCR	EMG responses		
1	M	L2-S2	Above L1	+	+	n.u	n. u	L1/2	− 1
2	M	Th10-L4	L5	n. u	n. u	+	L3-S3 right, L3/4 left	L1/2	3
3	M	L4-S1	S1	−	−	−	L3-S3	L5	1
4	F	Th10-L4	L4	InA	InA	InA	L3-S3	L1/2	3
5	F	S2-S4	S2	−	−	−	L3-S3	S3/4	1
6	M	S2-S3	S3	−	−	+	L3-S3	No deficit	2
7	F	L1-3	S3	+	+	+	L1-L3	L3	3

*n. u.* not used, *InA* inhalation anesthesia

Neurological examinations were performed immediately after birth by a pediatric neurosurgeon and neonatology team. Surgery was scheduled within 24 h, with one neurosurgeon (MK) performing the procedures using microsurgical techniques. The IONM protocol used for the neonates was identical to our well-established spinal monitoring protocol for lipoma and tethered cord surgery in children. IONM was employed throughout the length of surgery, including monitoring of MEP, SEP, and BCR. Free-running EMG recordings from L2-S2/3 nerve root muscles were intended to be used for anatomical orientation during placode resection with following bilateral muscle electrodes: M. adductor magnus femoris (L2), M. quadriceps femoris (L3-L4), M. tibialis anterior (L5), M. gastrocnemius (S1), M. abductor halluces (S2), and M. sphincter ani (S2-S3). SEP was measured for the median and tibial nerve.

Furthermore, EMG was intended to identify functional motor rootlets and nonfunctional caudal parts of the placode during placode release and neurulation. We intended also to monitor undue mechanical stress during surgical manipulation by IONM.

The anatomical level of the ONTD was assessed using fetal MRI performed 1–2 days before scheduled cesarean section and confirmed by postnatal MRI at 12 months. The ONTD level was defined as the first spinal segment without posterior osseous coverage, counted from C1. Follow-up evaluations were conducted at an age of 2 years in the outpatient spina bifida center.

This study was approved by the local ethics committee (Ethikkommission Universität Leipzig Az 330–13–18112013).

## Results

IONM was able to yield useful signals either by EMG and/or SEP and MEP measurements in all seven patients during postnatal ONTD repair. Table 1 provides an overview of patient characteristics, anatomical levels, and IONM results. Due to inhalation anesthesia, the cause of missing SEP, MEP, and BCR responses in patient #4 could not be differentiated between iatrogenic and neurological. However, direct EMG was possible in that patient. In the remaining 6 of 7 patients that underwent total intravenous anesthesia (TIVA), direct EMG responses were normal. MEP responses were detected in only 33% of patients. SEP monitoring at the tibial nerve was normal in 2 of 6 patients. BCR was implemented in 6 patients and yielded useful responses in 3 patients. That is, in three patients, BCR was reliably measured providing information about intact conus function at baseline before and during the ONTD repair procedure. In all other 3 patients, BCR response potentials were absent, or not clearly and repeatedly identifiable during baseline measurement before starting the surgical procedure.

No complications related to IONM occurred during surgery. Transient spontaneous EMG responses were observed during placode manipulation, but these normalized after pausing surgery. Thus, IONM helped to warn the surgeon when exerting undue stress to the placode and nerve roots during the procedure.

No significant changes in response patterns were observed during surgery. IOMN was also useful to identify individual functional motor nerve rootlets, to distinguish functional parts of the placode from aberrant parts esp. caudal to S2 and S3 motor roots.

Postoperatively, none of the patients experienced immediate neurological deficits. However, some patients showed fading tendon jerks in the early weeks following surgery. In patient #1, neurological function stabilized at the expected anatomical level with normal MEP and SEP responses. MRI revealed no postoperative cause for deterioration. Two patients (#5 and #6) with absent MEP and SEP responses remained functional without major motor or urological deficits at the 3-year follow-up.

At the final neurological examination, conducted up to 7 years post-surgery, 85% of the patients exhibited at least a one-level gain in motor function compared to their anatomical defect level. Only one patient with an anatomical MRI level of L2 presented with a level loss at the last outcome examination. There was no discrepancy in anatomical level evaluation between the fetal and postnatal follow-up MRIs at 12 months.

Comparing the postnatal neurological motor level assessment with the long-term motor outcome showed a much better motor level at the time of birth and a secondary decline between ONTD repair and outcome examination in 6 of 7 patients.

## Discussion

This is the first study, to our knowledge, that compares anatomical MRI levels with IONM results and 2-year motor outcomes following postnatal ONTD repair. Intraoperative neuromonitoring demonstrated intact EMG function of nerve roots in almost all patients, even in those with high anatomical defect levels. This underscores the value of IONM in preserving neurological function during surgery. Combining the different modalities of IONM, we were able to detect undue mechanical stress during surgical manipulation to the very delicate structure in the newborn in every patient. We postulate that we might have missed such events without monitoring.

No significant intraoperative complications occurred, and postoperative function stabilized in most patients, although some degree of early neurological deterioration was observed. This mirrors findings from Brune et al., who also reported significant functional losses following prenatal MMC surgery at lower spinal levels [6]. Interestingly, our small cohort showed improved functional levels in 85% of patients, compared to the expected anatomical level of MRI. However, the significant secondary decline after ONTD repair warrants further investigation on beneficial treatments to prevent it. Postoperative scarring, fibrosis, and neurodegeneration might be potential cascades that are worth to be investigated but lay far beyond the scope of the investigation.

The study has several limitations. The small sample size limits the generalizability of these findings. Additionally, the availability of IONM and the primary surgeon (MK) during unscheduled deliveries was a factor that constrained the sample. Nonetheless, our findings support the value of IONM in improving surgical safety. IONM supported careful manipulation and placode tailoring without sacrificing intact nerve rootlets, esp. to pause surgery in case of spontaneous EMG activity. Positive MEP, SEP, and BCR responses yielded additional safety for neurulation of the placode and dural closure by stable signals during all steps of surgery until the end of the procedure. Thus, trying to establish evoked potentials in MMC surgery is safe and—if positive—can add further safety to prevent intraoperative neural damage. Whether the use of IONM in postnatal ONTD repair surgeries may contribute to a “better-than-expected” long-term motor outcome compared to “traditional” postnatal surgery needs to be investigated in larger prospective studies. Also, it remains unclear, whether intraoperative EMG may have a role in predicting the long-term motor outcome of ONTD patients.

We hypothesize that the positive EMG responses across all motor nerve roots in these patients are indicative of more favorable functional outcomes. This aligns with the findings of Eibach and Pang, who demonstrated that intraoperative EMG during resection of dysfunctional parts of the placode also provided good postoperative results [5]. However, because of the current paucity of data, this remains a mere hypothesis to be carefully investigated.

Postnatal ONTD repair should evolve towards standardization, particularly regarding IONM use. Centralizing these surgeries in specialized centers might help reduce postoperative complications and optimize neurological outcomes [7]. Standardized follow-up care is essential for comparing data between pre- and postnatal surgery programs.

## Conclusion

The use of IONM during postnatal ONTD repair is safe and offers significant potential for improving surgical safety. Positive EMG responses were detected in most patients, even at higher defect levels. By 2 years of age, most patients demonstrated better motor function than expected based on anatomical MRI findings. These results advocate for further investigation of IONM in larger postnatal cohorts to evaluate whether intraoperative EMG may also be a useful parameter for prognostication.

## Data Availability

No datasets were generated or analysed during the current study.
